# Differences in photosynthetic responses of NADP-ME type C4 species to high light

**DOI:** 10.1007/s00425-016-2632-1

**Published:** 2016-12-18

**Authors:** Elżbieta Romanowska, Alicja Buczyńska, Wioleta Wasilewska, Tomasz Krupnik, Anna Drożak, Paweł Rogowski, Eugeniusz Parys, Maksymilian Zienkiewicz

**Affiliations:** Department of Molecular Plant Physiology, Faculty of BiologyUniversity of Warsaw, Miecznikowa 1, 02-096 Warsaw, Poland

**Keywords:** Bundle sheath and mesophyll chloroplasts, Fluorescence, Photosynthesis rate, Protein phosphorylation, Photosystems activity, Photoinhibition

## Abstract

**Main conclusion:**

**Three species chosen as representatives of NADP-ME C4 subtype exhibit different sensitivity toward photoinhibition, and great photochemical differences were found to exist between the species. These characteristics might be due to the imbalance in the excitation energy between the photosystems present in M and BS cells, and also due to that between species caused by the penetration of light inside the leaves. Such regulation in the distribution of light intensity between M and BS cells shows that co-operation between both the metabolic systems determines effective photosynthesis and reduces the harmful effects of high light on the degradation of PSII through the production of reactive oxygen species (ROS).**

**Abstract:**

We have investigated several physiological parameters of NADP-ME-type C4 species (e.g., *Zea mays, Echinochloa crus*-*galli*, and *Digitaria* *sanguinalis*) grown under moderate light intensity (200 µmol photons m^−2^ s^−1^) and, subsequently, exposed to excess light intensity (HL, 1600 µmol photons m^−2^ s^−1^). Our main interest was to understand why these species, grown under identical conditions, differ in their responses toward high light, and what is the physiological significance of these differences. Among the investigated species, *Echinochloa crus*-*galli* is best adapted to HL treatment. High resistance of the photosynthetic apparatus of *E. crus*-*galli* to HL was accompanied by an elevated level of phosphorylation of PSII proteins, and higher values of photochemical quenching, ATP/ADP ratio, activity of PSI and PSII complexes, as well as integrity of the thylakoid membranes. It was also shown that the non-radiative dissipation of energy in the studied plants was not dependent on carotenoid contents and, thus, other photoprotective mechanisms might have been engaged under HL stress conditions. The activity of the enzymes superoxide dismutase and ascorbate peroxidase as well as the content of malondialdehyde and H_2_O_2_ suggests that antioxidant defense is not responsible for the differences observed in the tolerance of NADP-ME species toward HL stress. We concluded that the chloroplasts of the examined NADP-ME species showed different sensitivity to short-term high light irradiance, suggesting a role of other factors excluding light factors, thus influencing the response of thylakoid proteins. We also observed that HL affects the mesophyll chloroplasts first hand and, subsequently, the bundle sheath chloroplasts.

## Introduction

In the leaves of C4 plants, the photosynthetic apparatus is partitioned between two cell types that are anatomically and biochemically distinct: bundle sheath (BS) and mesophyll (M) cells. These cells differ in their morphology as well as in the structure and specialization of their thylakoid membranes (Hatch 1987; Friso et al. 2010). Based on the differences of the major decarboxylation enzymes present in BS cells, C4 plants are classified into three biochemical subtypes: NADP-ME, NAD-ME, and PEP-CK (Hatch et al. 1975; Sheen 1999), where the NADP-ME species possesses structurally dimorphic chloroplasts. M chloroplasts are similar to those reported for other species of higher plants, whereas BS chloroplasts are characterized by highly reduced grana and various amounts of photosystems, and their photochemical activity, which may be related to the species or the habitat of these plants (Furbank and Foyer 1988). It is known that maize bundle sheath chloroplasts have the most reduced granal stacking (Taniguchi et al. 2003). Their level of photosystem II (PSII) in BS is relatively low and they contain mostly photosystem I (PSI). Although the PSII in BS membranes contains all the polypeptides involved in electron transport and oxygen evolution, it is practically inactive (Romanowska et al. 2006, 2008; Drożak and Romanowska 2006). Earlier, it was suggested that the agranal BS chloroplasts of maize are typical for the genus and do not appear to be influenced by irradiance (Downton and Pyliotis 1971). There have been also other reports on the functional PSII in bundle sheath chloroplasts of maize, claiming up to 50% of the whole-chain electron transport capacity seen in the thylakoids of C3 plants (Hardt and Kok 1978; Walker and Izawa 1979). It was also demonstrated that the level of the PSII in maize is regulated by the synthesis of key core components (Meierhoff and Westhoff 1993), and it has been postulated that the PSII content of the BS chloroplasts may be proportional to the amount of the aspartate transported from mesophyll (Chapman and Hatch 1981; Meister et al. 1996). Pengelly et al. (2010) suggested that an increased PSII content observed in both M and BS chloroplasts of *Flaveria bidentis* in low light might be due to the adaptation of PSII to growth light irradiance. Regulation of PSII levels in BS chloroplasts has not yet been extensively examined; therefore, maize chloroplasts may be considered as an appropriate model to study the relationship between structure and function in thylakoid membranes affected by various environmental factors. The opposite examples to BS chloroplasts in maize could be other NADP-ME species, such as *Echinochloa crus*-*galli* and *Digitaria* *sanguinalis*, representing the scope of variation in abundance of grana in BS chloroplasts (Ueno et al. 2005). There is a lack of information about the effects of light conditions on photochemistry of M and BS chloroplasts of these species, hence they are an interesting system for comparative studies of the response of these chloroplasts in varying light conditions. Due to the differences observed in both M and BS chloroplasts function, specific mechanisms are required to adjust the photosynthetic activity, upon variation in light quality and intensity.

Chloroplasts are plant organelles that are most sensitive to various stress factors, and light is one of the most important factors promoting a specific rearrangement of the chloroplast machinery in response to changes in its intensity and quality. In previous studies, we have revealed that the response of C3- and C4-type plants depends on both light quality and quantity, which was confirmed by the differences observed in light saturation of the investigated chloroplasts according to the light-penetration profile of the leaves (Zienkiewicz et al. 2015). When a leaf is exposed to light intensities exceeding their photosynthetic capacity, a decrease in the yield of chlorophyll fluorescence occurs, as part of the loss of excitation energy in the form of heat (non-photochemical quenching, NPQ) (Krause and Weis 1991). An increase in the levels of NPQ, while exposed to light irradiation, is correlated to a decline in the quantum yield of photosynthesis (Genty et al. 1989). The component of NPQ responsible for transthylakoidal membrane proton gradient activates violaxanthin de-epoxidase, thereby increasing zeaxanthin formation which in turn is involved in fluorescence quenching. Presumably, the thermal dissipation of energy is the main way of deactivating PSII under high light conditions since photorespiration in C4 plants is inhibited. Because *Zea mays* has a smaller pool of xanthophyll cycle pigments compared to C3 plants, the non-radiative energy dissipation is not univocally dependent on the zeaxanthin content, and other photoprotective mechanisms may be involved under stress by high light irradiance (Brugnoli et al. 1994). It seems, therefore, that the antioxidant metabolism can be implicated in determining the sensitivity of NADP-ME-type C4 plants to light stress, and its precise mechanism is more complicated compared to C3 plants. The M and BS cells have different antioxidant capacity (Doulis et al. 1997; Pastori et al. 2000). Some enzymes such as catalase are equally distributed between the two cell types, whereas ascorbate peroxidase (APX) and superoxide dismutase (SOD) are mainly localized in BS cells (Foyer et al. 2002). Such partitioning of antioxidants in the M and BS cells can minimize the accumulation of active oxygen species and is essential for plant homeostasis. The intercellular specialization helps in the efficient operation of photosynthesis, in particular stress conditions. The exposure of plants to high light can limit the electron transport, increase photoinhibition, and decrease the photosynthetic efficiency associated with PSII damage (Ohnishi et al. 2005; Hakala et al. 2006; Nishiyama et al. 2006).

To minimize photodamage, plants have developed protective mechanisms, such as rapid movement of leaves and chloroplasts, reversible phosphorylation of the thylakoid proteins, or state transitions. Also changes in the LHC protein level and modification of the leaf structure, morphology, or composition may occur (Givnish 1988; Ruban 2009). State transitions are the principal types of responses to changes in light quality, which are different from light-harvesting regulation induced by changes in light intensity (Minagawa 2011), whereas photoinhibition is caused by an imbalance between the rates of the damage and repair cycle of photosystem II proteins in thylakoid membranes. Lack of Calvin cycle in M chloroplasts and much lower generation of NADPH in BS chloroplasts compared to M cells may disturb the redox status established between the two cells types, and depending on the metabolic conditions, they may also contribute to PSII photoinhibition (Furbank and Foyer 1988). Phosphorylation of several PSII core subunits and LHCII proteins depends on the light intensity and their interactions with kinases and phosphatases (Baena-Gonzalez et al. 1999; Pribil et al. 2010; Shapiguzov et al. 2010). The phosphorylation of core proteins depends on light intensity, which was confirmed by the increased level of phosphorylation observed in high light conditions (Baena-Gonzalez et al. 1999). Phosphorylation of D1 protein is implicated in the regulation of this protein degradation (Rintämaki et al. 1996). The regulation of the repair cycle of PSII in BS compared to M thylakoid membranes of high light-treated C4 plants is not yet well known (Pokorska et al. 2009). All acquired data come from the experiments performed on *Z. mays*, and it is unknown whether other NADP-ME species show the same response to HL. Phosphorylation of LHCII proteins is responsible for the redistribution of the absorbed light energy between two photosystems (Rintamäki et al. 2000). The reversible phosphorylation of LHCII regulates the relative rates of the cyclic and noncyclic electron transport in chloroplasts and, thereby, it balances the rates of synthesis of ATP and NADPH with the demands of the Calvin cycle and of other metabolic pathways operating within the organelle (Grieco et al. 2015). High ATP concentration in the chloroplasts increases protein phosphorylation, while simultaneously inhibiting phosphatases (Rintämaki et al. 1996). The ATP level can also affect the activity of chloroplast proteases (Lindahl et al. 2000). It remains largely unknown to what extent the C4 plants coordinate the energy distribution between the M and BS chloroplasts, associated with response to changes in light intensity/quality.

The aim of this study was to investigate whether the chloroplasts of *Z. mays*, *D. sanguinalis*, and *E. crus*-*galli*, all NADP-ME-type C4 plants, possess the same mechanisms capable of inducing similar responses to high light conditions, thereby maintaining high photosynthetic efficiency. The question regards the mechanism by which the NADP-ME plants maintain the light reaction with maximal efficiency under photoinhibitory light treatment. For the first time, we reported that three NADP-ME species cultivated under the same conditions differ in their photosynthetic and respiratory responses to high light treatment. Our results indicated that out of the three investigated species, *E. crus*-*galli* was the most impervious to high light (HL) treatment, and possible reasons for its higher tolerance toward photoinhibitory light treatment are discussed. High resistance of photosynthetic processes to HL in *E.crus*-*galli* plants positively correlates with the phosphorylation level of PSII protein subunits, higher values of photochemical quenching and ATP/ADP ratio, organization of PSI and PSII complexes, as well as integrity of the thylakoid membranes.

These data provide a novel insight into the relations between differential membrane structure and function in thylakoids of M and BS chloroplasts of the NADP-ME-type species and show different mechanisms preventing photoinhibition. We believe that some of our observations may serve as a baseline for further exploration of the C4 photosynthetic metabolism important for the introduction of the C4-type pathway into the C3-type plants such as rice in order to maximize their productivity.

## Materials and methods

### Plant material and growth conditions

Maize seeds (*Z. mays* L.) were provided by the Plant Breeding and Acclimatization Institute (IHAR)-National Research Institute (Radzików, Poland); seeds of *D. sanguinalis* and *E. crus*-*galli* were collected from plants growing in the field near Warsaw (Walendów, Poland). NADP-ME-type C4 plants: *Z. mays* was grown in vermiculite, whereas *D. sanguinalis* and *Echinochloa crus*-*galli* plants were grown hydroponically in aerated Knop’s solution. Maize plants were fertilized with Knop’s solution containing (g L^−1^) 0.8 CaNO_3_·4H_2_O, 0.2 KNO_3_, 0.2 KH_2_PO_4_, 0.2 MgSO_4_·7H_2_O, and 0.028 EDTA-Fe enriched with A–Z microelement nutrients. Plants were grown in a growth chamber with a 14 h long irradiation photoperiod and a day/night regime at 24/21 °C at an irradiance of 200 µmol photons m^−2^ s^−1^ (ML). Detached leaves from plants were placed in water and transferred to high light (HL, 1600 µmol photons m^−2^ s^−1^) for 1 h at 25 °C prior further measurements and thylakoid isolation. Only photosynthesis rate (Pn) was measured every 20 min throughout 2 h in the leaves previously illuminated by photoinhibitory light (HL). Leaves were harvested from 4- to 5-week-old plants.

### Chloroplast and thylakoids isolation

Mesophyll and bundle sheath chloroplasts were isolated mechanically according to the procedure by Romanowska et al. (2006). Isolation procedures were carried out at 4 °C, under dim green light. The isolation buffers were supplemented with 10 mM NaF as a phosphatase inhibitor. Thylakoids were prepared by washing M and BS chloroplasts in a medium containing 50 mM Hepes–NaOH (pH 7.5), 5 mM MgCl_2_, 10 mM NaCl, 2 mM EDTA, 10 mM NaF, and collected by centrifugation at 8000*g* for 15 min. Isolated thylakoid samples were immediately frozen in liquid nitrogen and stored at −80 °C until use. The purity of chloroplasts was monitored by measuring the chlorophyll *a*/*b* ratio, enzymatic assays, and immunodetection of selected proteins, as described in Romanowska and Parys (2010).

### Measurements of PSI and PSII electron transfer activity

PSII activity of M and BS thylakoids of *Z. mays*, *D. sanguinalis*, and *E. crus*-*galli* was monitored spectrophotometrically as dichlorophenol–indophenol (DCPIP) photoreduction as described in Mamedov et al. (2000). Photoreduction was measured as the decrease in absorbance at 590 nm in a medium containing 330 mM sorbitol, 40 mM Hepes–KOH (pH 7.6), 1 mM KH_2_PO_4_, 5 mM NaCl, 5 mM MgCl_2_, 5 mM NH_4_Cl, and 0.1 mM DCPIP. The applied light was at an intensity of 1000 µmol photons m^−2^ s^−1^. Earlier, we had found similar PSII activities for the light irradiances in the range 600–3000 µmol photons m^−2^ s^−1^ (Pokorska and Romanowska 2007). The absorbance was read at 30 s intervals during the 1-min assays. The total volume of the reaction mixture was 2 ml including the chloroplast sample of 20 µg Chl.

PSI activity was measured using a Clark oxygen electrode (TriOximatic EO200; WTW, Weilheim, Germany). After 3 min of adaptation of the isolated thylakoids in the dark, activity was measured at 25 °C at 1000 μmol photons m^−2^ s^−1^. The total volume of 2 mL of the reaction mixture contained 50 mM Hepes–KOH (pH 7.7), 100 mM sorbitol, 2 mM EDTA, 1 mM KCl, and 5 mM MgCl_2_. PSI activity was measured as oxygen uptake in the reduction reaction of 0.2 mM TMPD (tetramethyl-*p*-phenylene diamine) with 3 mM ascorbate as the electron donor and 0.1 mM methylviologen (MV) as the final electron acceptor; 10 mM 3-(3,4-dichlorophenyl)-1,1-dimethylurea (DCMU) and 5 mM NaN_3_ were used as the PSII and catalase inhibitor, respectively. For electron transport measurements, chloroplast suspension containing 30 μg of chlorophyll was added to the reaction mixture.

All results are presented as mean values ± standard error (SE). The significant differences between means were determined using Student’s *t* test for paired observations at a confidence level of *α* = 0.05.

### SDS-PAGE and immunodetection analysis

Denaturing SDS-polyacrylamide gel electrophoresis was carried out according to Laemmli (1970). Thylakoid samples were diluted in the denaturing buffer (1/1, v/v) containing 0.25 M Tris–HCl (pH 6.8), 4% (w/v) SDS, 10 M urea, 2% (v/v) 2-mercaptoethanol, and 20% (v/v) glycerol. Separating gel (15% acrylamide) contained 6 M urea. Gels were loaded with thylakoid samples on an equal Chl basis. For immunoblotting analysis, the proteins resolved by SDS-PAGE were electrotransferred onto a PVDF membrane (Millipore, Badford, MA, USA) using 25 mM Tris, 192 mM glycine (pH 8.3), and 10% (v/v) methanol in the transfer buffer, as described by Towbin et al. (1979). The blocking was done with 5% fatty acid-free albumin (Sigma-Aldrich). Blots were probed with D1 (Agrisera, Vännäs, Sweden, AS05 084) and antiphosphotreonine (anti-P-Thr) antibodies (Cell Signalling Technology, #9381). The membranes were incubated with horseradish peroxidase-conjugated secondary antibody (Agrisera, Vännäs, Sweden, AS09 602), and cross-reacting protein bands were visualized by enhanced chemiluminescence according to the standard procedures using Chemi Doc System (Bio-Rad).

### Blue native (BN)-PAGE

Protein solubilization and BN-PAGE were performed according to Schägger (2001) and Kügler et al. (1997) with slight modifications. Thylakoid membranes (45 µg Chl) were sedimented at 7000*g* for 5 min at 4 °C and resuspended in a buffer composed of 5 mM 6-aminohexanoic acid (EACA), 50 mM imidazole–HCl (pH 7.0), 50 mM NaCl, and 0.5 mM EDTA. Membrane proteins were solubilized by the addition of *n*-dodecyl β-d-maltoside (DDM) to a final concentration of 1% for M and 2% for BS thylakoids (w/v). Samples were incubated on ice for 5 min and centrifuged at 15,800*g* for 40 min. The supernatant was supplemented with Coomassie Brilliant Blue solution (100 mM EACA, 30% sucrose) to a final concentration of 10% and loaded directly onto 4–10% acrylamide and 4–15% sucrose gradient gel. Electrophoresis was carried out using constant voltage of 100 V at 4 °C. The results shown on the gels are representative of those obtained in at least three independent experiments.

### Measurements of CO_2_ exchange

The CO_2_ exchange was measured as described earlier (Romanowska et al. 2002; Parys et al. 2004) using infrared CO_2_ analyzer (Beckman 865). The middle segments of leaves were placed into a plexiglas photosynthetic chamber in a bag containing water. Temperature was 22 ± 1 °C during the dark and light periods. Respiration rate before photosynthesis (*R*_0_) was determined in leaves adapted to darkness for 10 h. Then, the leaves were exposed to light (400 μmol photons m^−2^ s^−1^) and photosynthesis (Pn) was measured. The respiration following photosynthesis (*R*_1_) was determined after 2–3 min of darkness, which represents the light-enhanced dark respiration (LEDR). Pn was also measured every 20 min throughout 2 h in the leaves previously illuminated by photoinhibitory light (1 h of 1600 μmol photons m^−2^ s^−1^, HL). The photosynthesis and respiration rates were calculated from the changes in CO_2_ concentration in the range 360–300 μL L^−1^. The rates of photosynthesis and respiration were expressed in relation to leaf area.

### In vivo measurement of chlorophyll a fluorescence

Simultaneous measurements of photosynthesis and Chl *a* fluorescence of the same part of the leaf of *Z. mays*, *D. sanguinalis*, and *E. crus*-*galli* were performed at room temperature with a fluorometer (FMS 1, Hansatech, UK) run by Modfluor software. Leaves were adapted to the dark for 30 min prior to measurements of maximum quantum efficiency of PSII photochemistry (*F*v/*F*m), and then they were irradiated with a saturating light of 4500 μmol photons m^−2^ s^−1^. Photochemical quenching (qP) and non-photochemical quenching (NPQ) were also measured according to the procedure of Genty et al. (1989) using actinic irradiation of 400 and 1020 μmol photons m^−2^ s^−1^. The standard amber-modulating beam had a center frequency of 594 nm. Fluorescence parameters were assayed for the leaves from growth conditions (ML) and after transffering them from ML to high light (HL) for 1 h.

In addition, the relative electron transport rates (ETR) were measured in the light, using a pulse amplitude modulated (PAM) fluorometry (DUAL-PAM-100 system Walz, Effeltrich, Germany) in the leaves of plants grown under ML and after 1 h high light (HL) treatment. ETR was measured as a function of irradiance. The duration of the irradiance pulse at each level of actinic light was set to 60 s. All results are represented as mean ± SE of 10 independent series of experiments (6–8 measurements each).

### Determination of ATP content in whole leaves

The leaf samples were prepared as described by Romanowska et al. (2002). The leaves were cut and immediately ground in liquid nitrogen. The powder obtained was treated with 10% (v/v) HClO_4_ and left for 5 min on ice. The ice-cooled samples were centrifuged at 10,000*g* for 2 min and aliquots of the supernatants were brought to pH 7.0 by adding 1 M triethanolamine in 5 M KOH. After 30 min on ice, the precipitated KClO_4_ was pelleted (10,000*g* for 2 min), and the adenylate contents were measured in the supernatants. ATP was determined by the firefly luciferase method (Gardeström and Wigge 1988). ADP was converted to ATP by pyruvate kinase (Boehringer, Mannheim, Germany) and determined as above. Each measurement was calibrated with an addition of ATP standard. The measurements were repeated at least three times in three to four separate experiments.

### Carotenoid analysis

Analytical high-pressure liquid chromatography (HPLC) was performed according to the modified method of Krupnik et al. (2013) using a maximum flow rate of 0.6 mL min^−1^ and a Nucleosil 100 C18 column (Teknokroma, Barcelona, Spain). Pigments were extracted twice from the isolated thylakoid samples (0.5 mg Chl) with 1 ml of acetone:methanol (7:2, v/v) mixture. The volume of the thylakoid membrane suspension was no greater than one-fourth of the extraction mixture. Cellular and protein debris was removed by centrifugation at 4 °C (10,000*g* for 10 min). The extract was concentrated in a SpeedVac at 30 °C to Chl *a* concentration of 1 mg mL^−1^. Concentrated samples (20 µg Chl) were loaded onto the C18 column, previously equilibrated with phase A (acetonitrile:water:triethylamine, 90:10:0.1, by vol.). Pigments were separated in a linear gradient of 10–60% of phase B (ethyl acetate). Chlorophylls and carotenoid pigments were detected by absorbance at 436 nm. The content of each carotenoid species was expressed as a ratio of the area underneath the pigment-corresponding peak to the area under the Chl *a* peak. The amounts of each carotenoid were estimated using the extinction coefficient proposed by Davies (1976). Pigment molar ratios (in acetonitrile) were calculated using extinction coefficients 134.7 mM^−1^ cm^−1^ for neoxanthin, 122.9 mM^−1^ cm^−1^ for violaxanthin, 128.3 mM^−1^ cm^−1^ for antheraxanthin, 133.3 mM^−1^ cm^−1^ for zeaxanthin, 136.1 mM^−1^ cm^−1^ for lutein, 56 mM^−1^ cm^−1^ for Chl *b*, 91.7 mM^−1^ cm^−1^ for Chl *a*, and 134.6 mM^−1^ cm^−1^ for β-carotene.

### Other determinations

Total chlorophyll content was determined in 80% (v/v) acetone according to Porra (2002). Protein was assayed according to Bradford (1976). The activity of ascorbate peroxidase (APX) and superoxide dismutase (SOD), and the amount of hydrogen peroxide (H_2_O_2_) and malondialdehyde (MDA) were assayed as described by Parys et al. (2014).

## Results

NADP-ME plants *Z. mays*, *D. sanguinalis*, and *E. crus*-*galli* were grown under moderate light (ML, 200 µmol photons m^−2^ s^−1^) and their responses to photoinhibitory light (HL, 1600 µmol photons m^−2^ s^−1^) were studied. Fluorescence in vivo, gas exchange, and ATP/ADP ratio of the leaves were measured.

### Effect of HL treatment on photosynthesis, respiration, and Chl *a* fluorescence in vivo

To determine changes in the short-term response of photosynthesis, characteristics of photosynthesis, respiration, and fluorescence of the leaves from three species growing in ML and following transferring to HL were measured.

**Fig. 1:**
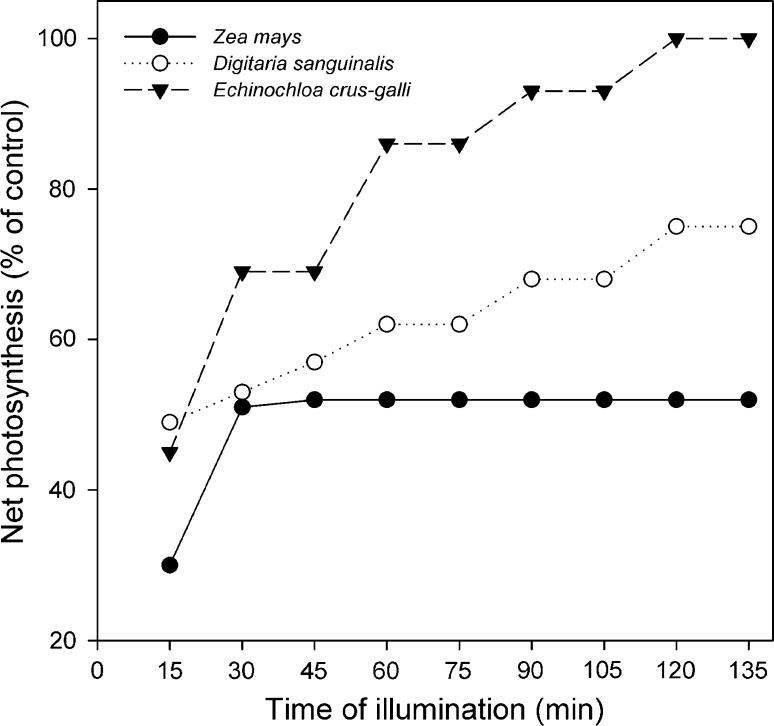
Photosynthesis rates of detached leaves of *Zea mays*, *Digitaria sanguinalis*, and *Echinochloa crus*-*galli* plants growing in moderate light (ML) and after transferring from ML to high light (HL) for 1 h. Photosynthesis was measured during 2 h of recovery after HL treatment and is expressed as a percentage of the control value (in µmol CO_2_ m^−2^ s^−1^): *Z. mays* 13.22 ± 0.62; *E. crus*-*galli* 23.02 ± 0.64; *D. sanguinalis* 19.9 ± 0.96

Figure 1 shows the changes in the net photosynthesis (Pn) rate observed during a short-term (2 h) period after the NADP-ME plants were treated with high light intensity. Before HL treatment, the control leaves of *D. sanguinalis* and *E. crus*-*galli* showed higher photosynthesis rate of 50 and 75%, respectively, compared to *Z. mays*. After high light treatment, the photosynthesis rate in maize leaves decreased by about 70% compared to control leaves, and after a slight increase in light, the rate remained unchanged during the recovery period, reaching 50% of control value after 2 h. In *E. crus*-*galli* leaves, Pn rate decreased after photoinhibition by 55% and returned to the control level during the recovery period. In case of *D. sanguinalis*, Pn rate decreased by about 50% during photoinhibition, and after 2 h of recovery the rate was about 25% lower compared to the control leaves. The data obtained show that among the selected species, *E. crus*-*galli* was found to be the most resistant plant to photoinhibition.

**Fig. 2:**
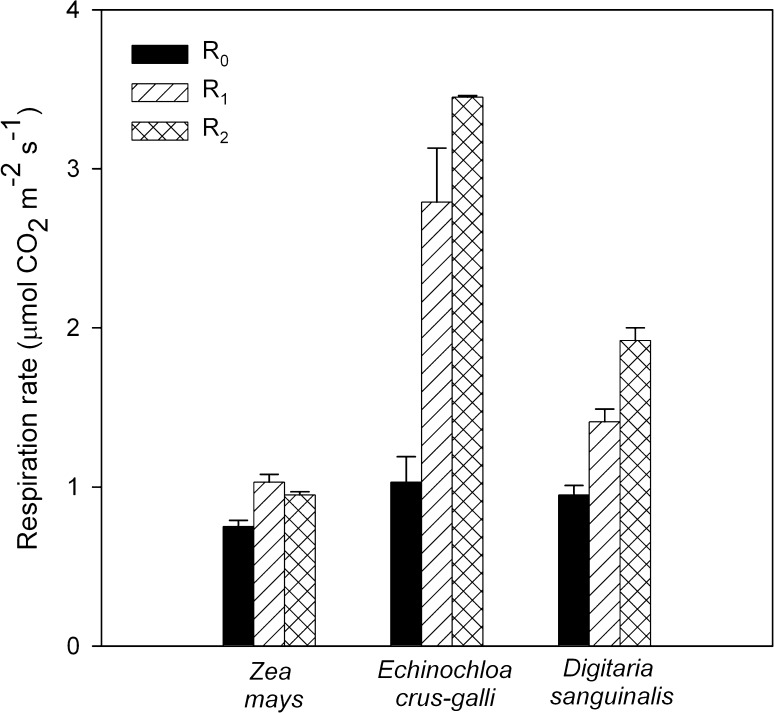
Respiration rates of detached leaves of *Zea mays, Echinochloa crus*-*galli*, and *Digitaria sanguinalis*.* R*_0_, respiration rate before photosynthesis was determined in leaves adapted to darkness for 10 h. Then, the leaves were exposed to light (400 μmol photons m^−2^ s^−1^) and photosynthesis (Pn) was measured. The respiration following photosynthesis (*R*_1_) was determined after 2–3 min of darkness, which represents the light-enhanced dark respiration (LEDR) of plants from growth condition (ML); *R*_2_, respiration measured 2 h after photoinhibitory light treatment (1600 μmol photons m^−2^ s^−1^). Values are the means of 4–5 separate experiments ± SE

The respiration rates of the NADP-ME species during growth conditions (ML) and following photoinhibition are shown in Fig. 2. Although the rate of Pn (Fig. 1) in *D. sanguinalis* was slightly lower than that in the *E. crus*-*galli* leaves, CO_2_ released during dark respiration following photosynthesis (*R*_1_) was different. *E. crus*-*galli* leaves showed a greater (about 100%) increase in the rate of *R*_1_ compared to *D. sanguinalis* leaves and about 2.7-fold higher compared to maize, although the respiration before illumination (*R*_0_) has been comparable for *D. sanguinalis* and *E. crus*-*galli*, and it was higher by about 25% than that in maize. When the plants were transferred to HL, the respiration rate after 2 h of recovery after HL (*R*_2_) increased by about 24 and 36% for *E.* *crus*-*galli* and *D. sanguinalis,* respectively. *R*_2_ in *Z. mays* was on the same level as after photosynthesis in ML. The photochemical efficiency of PSII (*F*v/*F*m) in the leaves of investigated NADP-ME plants (Table 1) was close to 0.8 in growth conditions, indicating that PSII is functional.

**Table 1:** Effects of photoinhibitory light on the maximal efficiency of PSII phytochemistry (*F*v/*F*m) in the dark-adapted leaves of *Echinochloa crus*-*galli*, *Digitaria sanguinalis*, and *Zea mays* in growth light conditions (ML) and after transferring leaves for 1 h to high light (ML → HL)

	*F*v/*F*m	
	ML	ML → HL
*Zea mays*	0.79 ± 0.004	0.53 ± 0.038
*Digitaria sanguinalis*	0.80 ± 0.010	0.65 ± 0.004
*Echinochloa crus*-*galli*	0.81 ± 0.007	0.75 ± 0.016

Data are mean ± SD of ten independent experiments

**Fig. 3:**
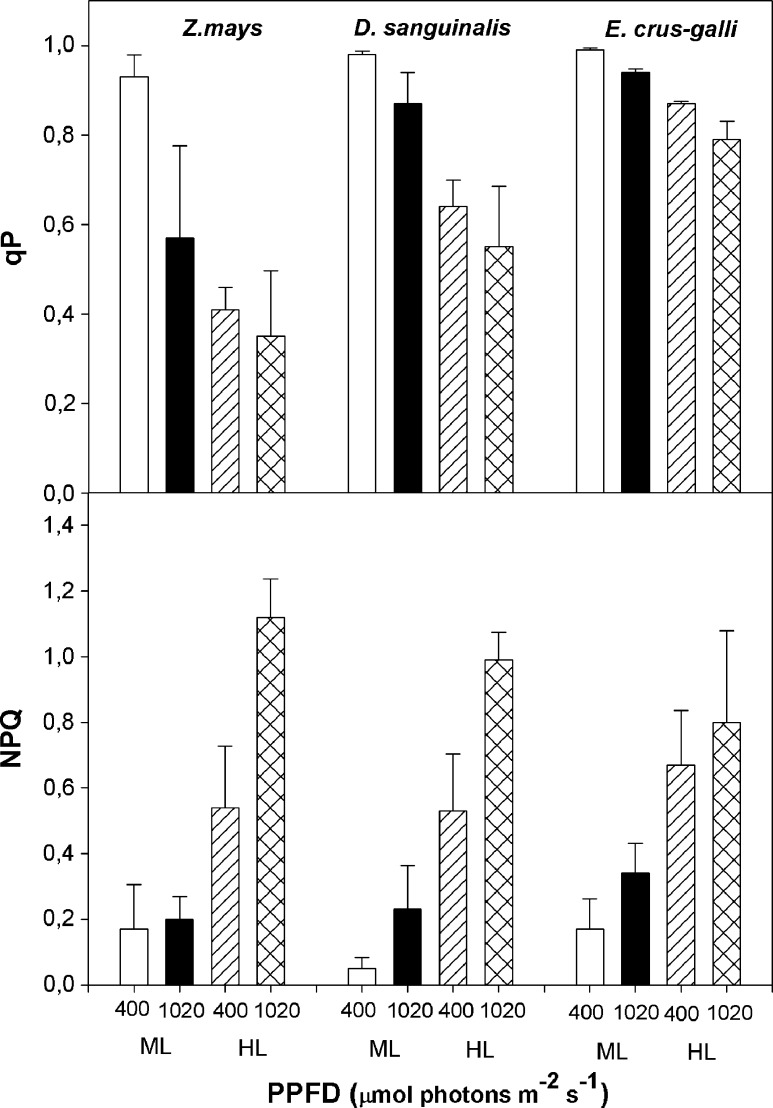
Responses of photosystem II photochemical (qP) and non-photochemical quenching coefficient (NPQ) to the actinic radiation (PPFD) of 400 and 1020 µmol photons m^−2^ s^−1^ in *Echinochloa crus*-*galli*, *Digitaria sanguinalis*, and *Zea mays* leaves of plants grown in ML and after treatment for 1 h with high light intensity (HL, 1600 μmol photons m^−2^ s^−1^). Data are means ± SE of 5–8 independent experiments

The *F*v/*F*m ratio is used for monitoring the in vivo effect of stress because it is linearly correlated with the quantum yield of light-limited O_2_ evolution. The *F*v/*F*m ratio measured after 1 h of photoinhibitory light treatment was dependent on the species, and it was lowest for maize (67% of the control value). In case of *E. crus*-*galli* and *D. sanguinalis* leaves, it decreased by about 8 and 19%, respectively (Table 1). Photochemical fluorescence quenching (qP) in the leaves of *E. crus*-*galli*, *D.sanguinalis*, and *Z. mays* grown under moderate light (ML) was similar when light intensity treatment was 400 μmol photons m^−2^ s^−1^, whereas at 1020 μmol photons m^−2^ s^−1^, qP declined by about 55 and 35% in *Z. mays* and *D. sanguinalis* and slightly in *E. crus*-*galli* (Fig. 3). After photoinhibitory light treatment, maximal reduction in qP was observed in maize leaves at PPFD of 400 and 1020 μmol photons m^−2^ s^−1^. High light in *E. crus*-*galli* resulted in a slight decrease in qP values in both PPFD treatments. In case of *D. sanguinalis* leaves, the inhibitory effect of HL treatment on qP was higher compared to *E. crus*-*galli* leaves. In these plants, thermal dissipation of excitation energy, as expressed by non-photochemical fluorescence quenching of chlorophyll (NPQ), increased after HL treatment in all investigated species. A highest increase was observed for maize leaves at 1020 μmol photons m^−2^ s^−1^ and it was about two times higher compared to 400 μmol photons m^−2^ s^−1^. In the same conditions, for *E. crus*-*galli*, the NPQ was very similar after HL treatment at 400 and 1020 μmol photons m^−2^ s^−1^, but for *D.* *sanguinalis*, the NPQ value was lowest at 400 μmol photons m^−2^ s^−1^.

**Fig. 4:**
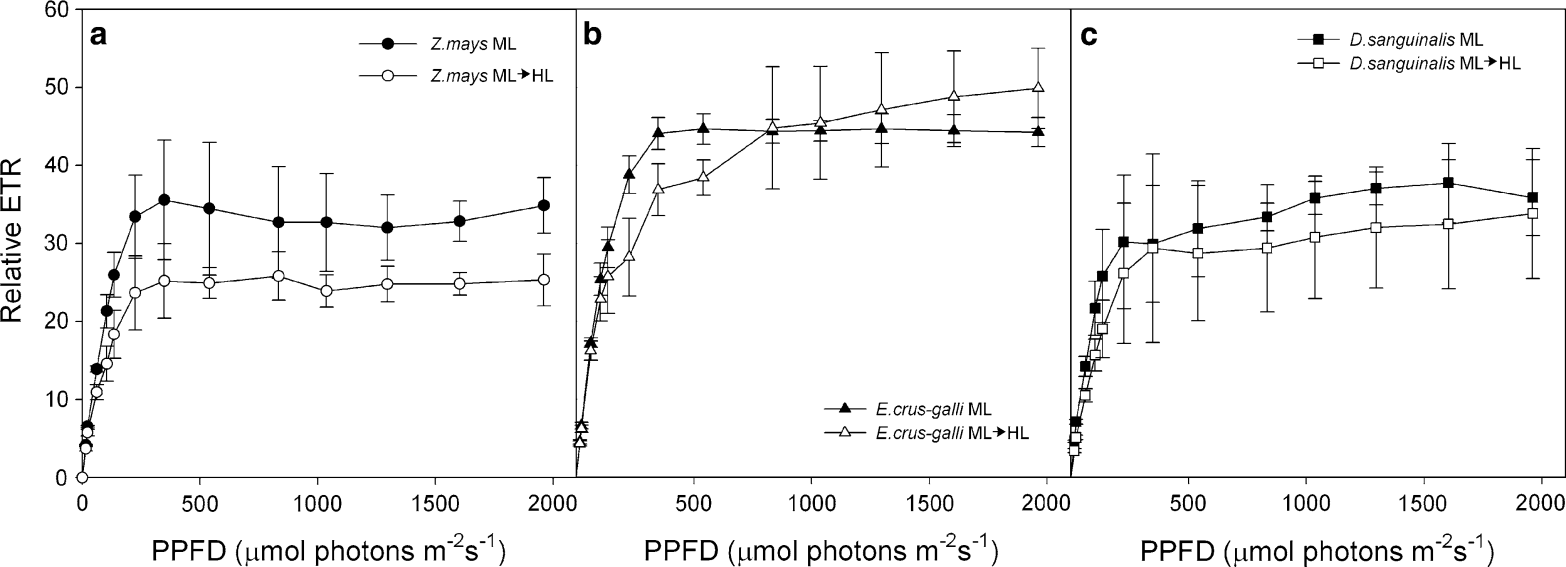
The typical courses of relative electron transport rate (ETR) of *Zea mays* (**a**) *Echinochloa crus*-*galli* (**b**) and *Digitaria sanguinalis* (**c**) leaves of plants grown in ML and after treatment for 1 h with high light intensity (ML → HL). ETR was measured as function of irradiance. The duration of irradiance at each level of actinic light was set to 60 s

Light response curves have provided detailed information on the electron transport capacity and limitation of PSII. PAM fluorometry was complemented by other methods, in which photosynthesis and respiration rates were measured. ETR was higher in the plants grown under ML than after HL treatment in the leaves of all investigated species. Significant rise of the electron transport rate (Fig. 4a–c) was observed at the lower intensities of the light curve (up to about 200 μmol photons m^−2^ s^−1^) and ETR was similar for ML and HL-treated species. In higher light intensities (above 540 μmol photons m^−2^ s^−1^), ETR did not increase significantly for all plants, but there were differences between species, as well as for the ML and the HL-treated leaves. The highest ETR was observed for *E. crus*-*galli* and it was similar in both ML and HL-treated leaves. In *Z. mays* the ETR, after HL treatment, decreased by about 27% at the light intensity range between 500 and 1961 μmol photons m^−2^ s^−1^, as compared with the ML, whereas in *D. sanguinalis* the HL inhibited ETR only slightly. The ETR after HL treatment was higher in *E. crus*-*galli* and then in *D. sanguinalis* and *Z. mays* by about 28% and 46%, respectively. The decrease in rates of ETR at high irradiances for Z. mays may occur due to the inhibition of CO_2_ assimilation in this plant. On the other hand, the increasing rates of ETR in *E. crus*-*galli* at >540 μmol photons m^−2^ s^−1^ may be due to a better adaptation of the BS cells to CO_2_ fixation.

### Effect of light treatment on the carotenoids composition in the M and BS thylakoids

The pigment composition in the *E. crus*-*galli*, *D. sanguinalis*, and *Z. mays* M and BS thylakoids isolated from the leaves of control plants (ML) and after 1 h of photoinhibitory light treatment (ML → HL) is shown in Table 2. The amount of photoconvertible V, expressed as a percentage of the V + A + Z pool in both M and BS thylakoids, isolated from the leaves of all investigated species after HL (1600 µmol photons m^−2^ s^−1^) treatment, displayed a similar pool of photoconvertible V. Usually, the sun-exposed leaves displayed higher photoconvertible V than shade leaves, similarly as in the C3 leaves compared to the C4 leaves. Similar levels of photoconvertible V did not correlate with the corresponding differences obtained due to the level of NPQ (Fig. 3). However, for *E. crus*-*galli* and *D. sanguinalis*, the differences in the levels of Z + A/Chl *a* under ML and after HL correlated well with the level of NPQ. The Z + A content increased after HL treatment significantly with one exception being the *D. sanquinalis* BS thylakoids, where exposure to ML and HL treatment yielded similar levels of Z + A. The total carotenoid contents (expressed on the basis of Chl *a*) were very similar in M and BS thylakoids for all species, with a small noticeable decrease in both M and BS thylakoids of *D. sanquinalis* and *Z. mays* compared to *E. crus*-*galli* (about 20%).

**Table 2:** Total carotenoids (lutein, neoxanthin, ß-carotene, violaxanthin (V), antheraxanthin (A), zeaxanthin (Z)) content; photoconvertible violoxanthin (V) and Z + A content in *Echinochloa crus*-*galli*, *Digitaria sanguinalis* and *Zea mays* M and BS thylakoids isolated from the leaves of control plants (ML) and after 1 h of photoinhibitory light treatment (ML → HL)

Parameters	*Zea mays*		*Digitaria sanguinalis*		*Echinochloa crus*-*galli*	
	M	BS	M	BS	M	BS
Total carotenoids (mol/100 mol Chl *a*)						
ML	27.4 ± 0.63	25.1 ± 0.84	24.9 ± 2.15	29.6 ± 0.56	33.6 ± 1.56	33.5 ± 1.41
ML → HL	28.0 ± 1.38	24.7 ± 1.38	26.3 ± 1.84	25.3 ± 1.89	30.9 ± 1.77	34.2 ± 0.48
Z + A (mol/100 mol Chl *a*)						
ML	0.8 ± 0.14	0.7 ± 0.07	1.1 ± 0.10	1.3 ± 0.15	0.8 ± 0.10	1.3 ± 0.21
ML → HL	2.8 ± 0.52	2.2 ± 0.39	2.0 ± 0.21	1.3 ± 0.13	2.8 ± 0.37	3.6 ± 0.32
Photoconvertible V (% of V + A + Z) after HL	60.0	63.4	64.3	71.0	60.1	57.9

Photoconvertible V is expressed on the basis of the V + A + Z pool. The data represent mean values ± SD obtained from 3 different measurements of one sample from a typical experiment

### PSII and PSI activities

PSII and PSI activities were measured in M and BS thylakoids isolated from the leaves of plants grown under ML and after transferring to HL (Table 3). During growth, an influence of light intensity on the activity of both photosystems in the investigated plants was observed. PSII in BS chloroplasts was less active than that in M chloroplasts. Some previous reports demonstrated that PSII was not active in BS chloroplast of maize or had low activity, whereas in *E. crus*-*galli* and *D. sanguinalis* it was about two times higher than that in maize. In M thylakoids, PSII activity was lower in *E. crus*-*galli* and *D. sanguinalis* as compared to maize, about 57 and 40%, respectively. Photoinhibitory light decrease of PSII activity reached about 50% in maize, whereas in *E. crus*-*galli* and *D. sanguinalis* it was only slightly diminished. In BS chloroplasts, HL treatment had a slight effect on PSII activity. Data of PSI and II activity measurements demonstrated that similar growth light conditions and inhibitory light induced various changes in M and BS chloroplasts of the investigated NADP-ME type C4 species. Large differences we observed between individual plants.

**Table 3:** PSI and PSII electron transport activity in isolated mesophyll (M) and bundle sheath (BS) thylakoids of *Echinochloa crus*-*galli*, *Digitaria sanguinalis*, and *Zea mays* chloroplasts from control plants (200 µmol photons m^−2^ s^−1^) and then transferred to high light (HL) for 1 h (1600 μmol photons m^−2^ s^−1^)

Light intensity (µmol photons m^−2^ s^−1^)	PSII activity (µmol DCPIP mg^−1^ Chl h^−1^)				PSI activity (µmol O_2_ mg^−1^ Chl h^−1^)			
	200		1600		200		1600	
	M	BS	M	BS	M	BS	M	BS
*Zea mays*	89 ± 1	15 ± 2	47 ± 1	10 ± 1	296 ± 1	1020 ± 9	156 ± 2	565 ± 3
*Digitaria sanguinalis*	40 ± 1	32 ± 3	37 ± 1	25 ± 9	284 ± 6	657 ± 1	176 ± 1	407 ± 4
*Echinochloa crus*-*galli*	57 ± 2	30 ± 7	49 ± 4	22 ± 9	189 ± 2	671 ± 2	156 ± 9	429 ± 5

Details are given in Materials and methods. The electron transport activities of PSI were measured polarographically at 25 °C. Chloroplast equivalents of 30 µg mL^−1^ (M) and 10 µg mL^−1^ (BS) of chlorophyll were used for PSI measurements, chloroplast equivalents of 20 µg mL^−1^ of chlorophyll were used for PSII measurements. DCPIP was 0.1 mM. Values are mean ± SD of three independent experiments for PSI, means of 4–5 separate experiments ± SE (three replicates each) for PSII

As expected, PSI activity was highest in maize BS chloroplasts and it was about 1.5-fold higher than that in *E. crus*-*galli* and *D. sanguinalis* chloroplasts. The PSI activity was approximately threefold higher in BS chloroplasts compared to M chloroplasts in maize grown in similar conditions as *E.* *crus*-*galli*. The PSI activity in BS chloroplasts of *D. sanguinalis* was 2.3 times higher than that in M chloroplasts. High light treatment decreased PSI activity in both types of maize chloroplasts by about 50%, in *D. sanguinalis* by about 40%, whereas in *E. crus*-*galli* by about 18 and 36% in M and BS chloroplasts, respectively, compared with the control chloroplasts (from plants grown under ML conditions).

The ATP/ADP ratio in the leaves of NADP-ME plants in darkness, growth conditions (ML) and after photoinhibition is shown in Table 4. The ATP/ADP ratio differed between light conditions and was highest in the ML for all three species. *E. crus*-*galli* leaves had also the highest observable values of ATP/ADP ratio. The ATP/ADP ratio dropped by at least 50–60% in the dark, as compared with growth conditions (ML) and it was also highest in *E. crus*-*galli*, in which dark respiration was highest (Fig. 2). Photoinhibitory light (ML → HL) caused the ATP/ADP ratio to decrease in leaves of all investigated species by about 16–20% as compared to ML. It was achieved by lower photosynthetic activity and, thus, lower respiration rate in these conditions (Figs. 1, 2).

**Table 4:** Effect of photoinhibition on the ATP/ADP ratio in the leaves of three selected species of NADP-ME grasses

	ATP/ADP		
	Darkness	ML	ML →HL
*Zea mays*	1.5	2.5	2.1
*Digitaria sanguinalis*	1.2	2.3	1.8
*Echinochloa crus*-*galli*	2.2	4.0	3.3

Leaves were harvested from plants grown at 200 μmol photons m^−2^ s^−1^ (ML) and then transferred to high light (HL, 1600 μmol photons m^−2^ s^−1^) for 1 h as well as after a dark period (10 h)

### PSII protein phosphorylation after photoinhibition

**Fig. 5:**
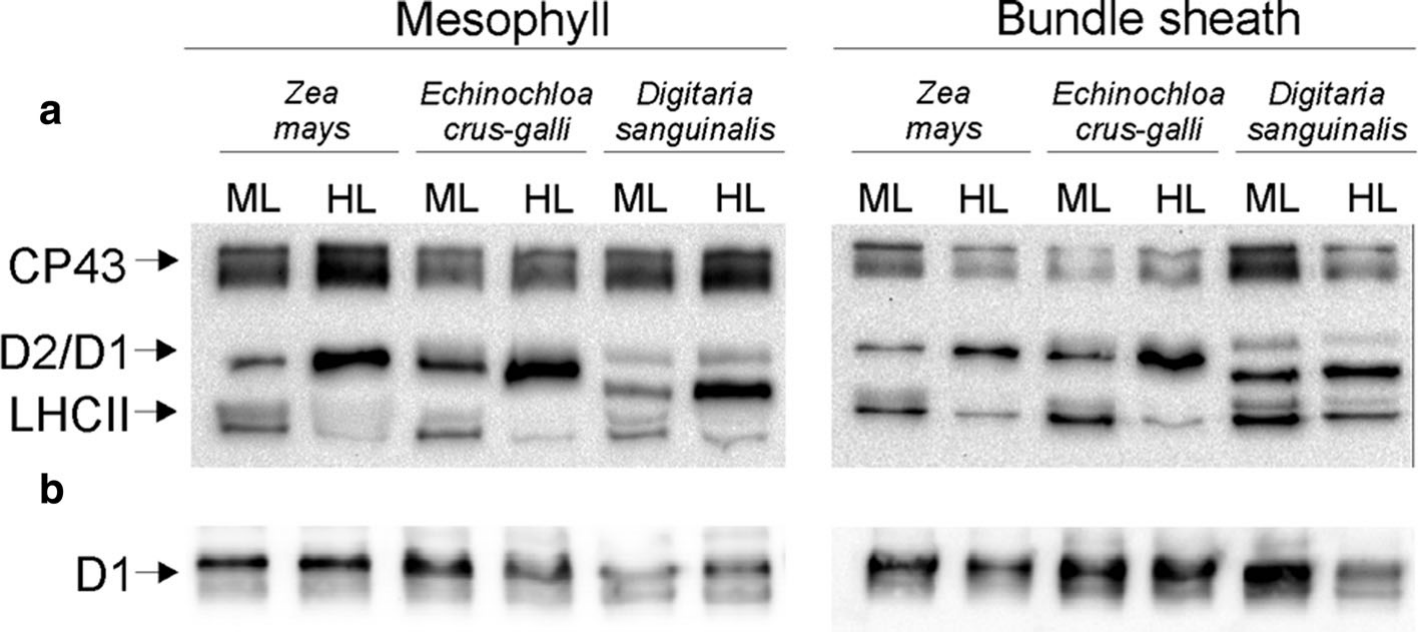
PSII protein phosphorylation in mesophyll and bundle sheath thylakoids isolated from the leaves of *Zea mays*, *Digitaria sanguinalis*, and *Echinochloa crus*-*galli.* The leaf samples were collected 2 h after growth light (ML) was turned on, and after that leaves were shifted for 1 h to high light (HL, 1600 μmol photons m^−2^ s^−1^) from ML (**a**). Gel wells were loaded with 1.5 µg of Chl equivalents of the isolated thylakoids. Phosphorylated proteins were detected with anti-PThr antibody. The positions of detected phosphoproteins are indicated on the left side. The level of D1 protein in thylakoids was confirmed using D1 protein-specific antibody (Agrisera) (**b**). Thylakoid proteins (1.0 µg Chl) were separated by urea SDS-PAGE and immunodetected. The results shown are representative of those obtained in at least three independent experiments. Thylakoids were isolated in the presence of 10 mM NaF

Modulations of PSII protein phosphorylation under photoinhibitory conditions in M and BS thylakoids isolated from control (2 h of ML illumination after night) and 1 h HL-treated leaves of NADP-ME grasses are shown in Fig. 5. Typically, low light conditions trigger only LHCII proteins phosphorylation, whereas phosphorylation of D1 protein is irradiance dependent. If similar light conditions differently influence protein phosphorylation in the investigated thylakoids, then we can assume that all investigated chloroplasts have different phosphorylation status. High light treatment of plant leaves increased the phosphorylation level of D1 in both M and BS thylakoids, as compared to ML controls (Fig. 5). The phosphorylation of D1 protein in M and BS thylakoids of *E. crus*-*galli* was higher than that in maize or in both types of thylakoids of *D. sanguinalis*. However, the phosphorylation of LHCII was higher in BS thylakoids than that in M thylakoids for all investigated plants, which may suggest that BS chloroplasts were exposed to lower light intensity compared to M chloroplasts, hence a low-light exposure phosphorylation pattern of LHCII was observed. In M chloroplasts, LHCII proteins were phosphorylated in ML and dephosphorylated in HL, suggesting light overexposure. These results indicate that differences in phosphorylation of PSII proteins might be related to light intensity that is effectively reaching the chloroplasts (M or BS) and/or their redox status in different species. Changes in phosphorylation of PSII proteins in maize M and BS thylakoids exposed to different light quality were observed already earlier (Zienkiewicz et al. 2015). These experiments clearly demonstrate that the same light conditions have different effect on phosphorylation of D1 and LHCII proteins in M and BS thylakoids among investigated plants, grown in the same light conditions. After HL treatment, the level of D1 protein in M and BS thylakoids remained at a similar level, despite elevated NPQ and lower PSII activity levels as compared to plants grown in ML.

### Abundance of M protein complexes in response to photoinhibitory light

**Fig. 6:**
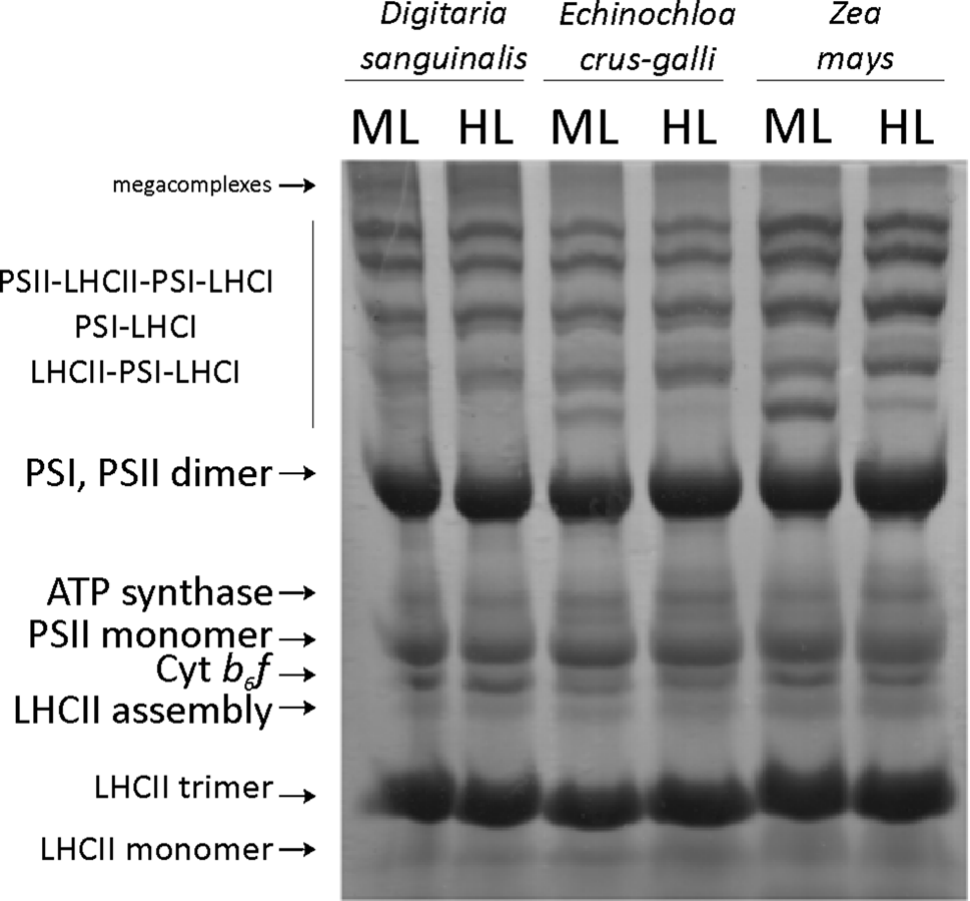
Blue native (BN)-PAGE. The composition of protein complexes in thylakoids isolated from mesophyll (M) chloroplasts of *Digitaria sanguinalis, Echinochloa crus*-*galli*, and *Zea mays.* Thylakoids were isolated from the leaves of plants grown at 200 μmol photons m^−2^ s^−1^ (ML) and from the leaves transferred to HL (1600 μmol photons m^−2^ s^−1^) for 1 h. Equivalents of 45 μg of Chl were loaded onto each lane. The identification of the complexes was done as described before (Mekala et al. 2015)

To compare the abundance of protein complexes during the high light stress in thylakoids of NADP-ME species, we employed blue-native (BN) electrophoresis. A typical pattern of the thylakoid membrane protein complexes is shown in Fig. 6. Thylakoids were isolated from control and HL-treated NADP-ME C4 plants. Under these conditions, 11 main protein bands, corresponding to thylakoid membrane complexes, were identified. The electrophoresis showed that the super-organization of thylakoid membrane proteins was not affected by short-term treatment with HL. The PSII and PSI complexes, together with various combinations of LHC, were resolved in 6 bands in ML and 5 bands in HL, and the remaining complexes did not show any differences between ML and HL. Bands representing the trimeric forms of the light-harvesting complex of PSII (LHCII) did not change significantly. Furthermore, no significant variations of the bands containing the cytochrome b_6_f and the ATP synthase complexes were observed. In this assay, the protein profiles obtained for control and HL-treated leaves were clearly similar. These data demonstrate that HL does not disturb the organization of thylakoid complexes. The preservation of the complexes cannot explain the observed decrease of photosystems activity upon increase of light intensity. The disappearance of the LHCII-PSI-LHCI complex indicates changing dynamics of the thylakoid complexes during short-term light changes. Interestingly, we did not observe any differences between M thylakoids of the investigated species.

### Effect of HL treatment on H_2_O_2_ and MDA content

The HL treatment affected the content of hydrogen peroxide (H_2_O_2_) and malondialdehyde (MDA) in the leaves of investigated C4 species (Table 5). After HL treatment, the content of MDA increased about 38, 32, and 88% for *Z. mays*, *E. crus*-*galli* and *D. sanguinalis* leaves, respectively, as compared to controls (ML). The MDA content in control and HL-treated maize leaves was higher than that in *E. crus*-*galli* and *D. sanguinalis* leaves. The level of the H_2_O_2_ was higher in maize leaves than that of *D. sanguinalis* and *E. crus*-*galli* grown in ML, by about 70 and 76%, respectively. The level of H_2_O_2_ in ML leaves was higher as compared to HL-treated leaves and it decreased by about 56% in maize and by about 23–25% in both *E. crus*-*galli* and *D. sanguinalis* leaves exposed to HL.

**Table 5:** Effects of photoinhibitory light (HL, 1600 µmol m^−2^ s^−1^) on H_2_O_2_ generation and lipid peroxidation measured as the malondialdehyde (MDA) content in the leaves of *Zea mays*, *Digitaria sanguinalis*, and *Echinochloa crus*-*galli* plants grown at 200 µmol photons m^−2^ s^−1^ (ML) and transferred to high light (ML → HL) for 1 h

	ML		ML → HL	
	(µmol g^−1^ * F*w)			
	H_2_O_2_	MDA	H_2_O_2_	MDA
*Zea mays*	0.93	11.9	0.52	16.5
*Digitaria sanguinalis*	0.29	7.4	0.22	13.9
*Echinochloa crus*-*galli*	0.22	8.5	0.17	11.2

The APX activity was highest in *E. crus*-*galli* leaves and it did not change after HL stress (Table 6). In *D. sanguinalis,* APX activity was lowest in growth condition (ML) and increased twofold during high light treatment. In *Z. mays* leaves, APX activity decreased by about 30% after HL treatment. SOD activity increased after HL treatment in *D. sanguinalis* (about twofold) and in *E. crus*-*galli* (25%), whereas in maize it was not changed.

**Table 6:** Effects of photoinhibitory light (HL, 1600 µmol m^−2^ s^−1^) on APX and SOD activity in the leaves of *Zea mays*, *Digitaria sanguinalis*, and *Echinochloa crus*-*galli* plants growing in 200 µmol photons m^−2 ^s^−1^ (ML) and transferred to high light (ML → HL) for 1 h

Parameters	*Zea mays*		*Digitaria sanguinalis*		*Echinochloa crus*-*galli*	
	ML	ML → HL	ML	ML → HL	ML	ML → HL
APX (µmol Asc min^−1^ mg^−1^ protein)	0.30 ± 0.05	0.21 ± 0.04	0.1 ± 0.01	0.2 ± 0.02	0.46 ± 0.07	0.48 ± 0.08
SOD (U mg^−1^ protein)	9.80 ± 1.1	10.1 ± 0.9	2.1 ± 0.4	4.7 ± 0.8	5.9 ± 0.4	7.4 ± 0.6

Data are mean ± SD (*n* = 3–5)

## Discussion

We have examined the effect of high light irradiation on photosynthesis, respiration and energetic status of leaves, and photochemical activities of mesophyll and bundle sheath thylakoids of the C4 NADP-ME species, in particular: *Z. mays, E. crus*-*galli* and *D.* *sanguinalis*. The light exposure variation in the environment is expected to alter the interaction between the functions of C4 plants chloroplasts due to the unequal distribution of excitation energy between M and BS chloroplasts (Tazoe et al. 2008). Little is known about the influence of light variation on the photochemical response of various species of NADP-ME C4 subtype. It also remains largely unknown, whether different mechanisms are required for adjustment of photosynthesis upon the induced changes in light quality and intensity in dependence on variation in the amounts of grana in BS chloroplasts. Ueno et al. (2005) have evaluated the granal index in BS chloroplasts of C3 and C4 grasses and ranked the degree of granal development in *Z. mays,* *D. sanguinalis* and in *E. crus*-*galli*, respectively, <3%, 5–10% and 15–25%. Its probably reflects the BS chloroplasts differences in the availability of the reducing power, which limits for CO_2_ fixation. In this paper, we set out to investigate the influence of the short-term effect of HL stress on the function of M and BS chloroplasts of the selected three species grown under medium light intensity (ML). It is unknown whether these species show the same response to photoinhibitory light. We compared the responses of the leaves and thylakoids to photoinhibitory light treatment with those resulting from control growth conditions (ML). We addressed the questions why do the NADP-ME C4 species respond differently to high light and what is the reason for the apparent photosynthetic differences in these plants? Is there a species-specific mechanism responsible for these effects?

**Fig. 7:**
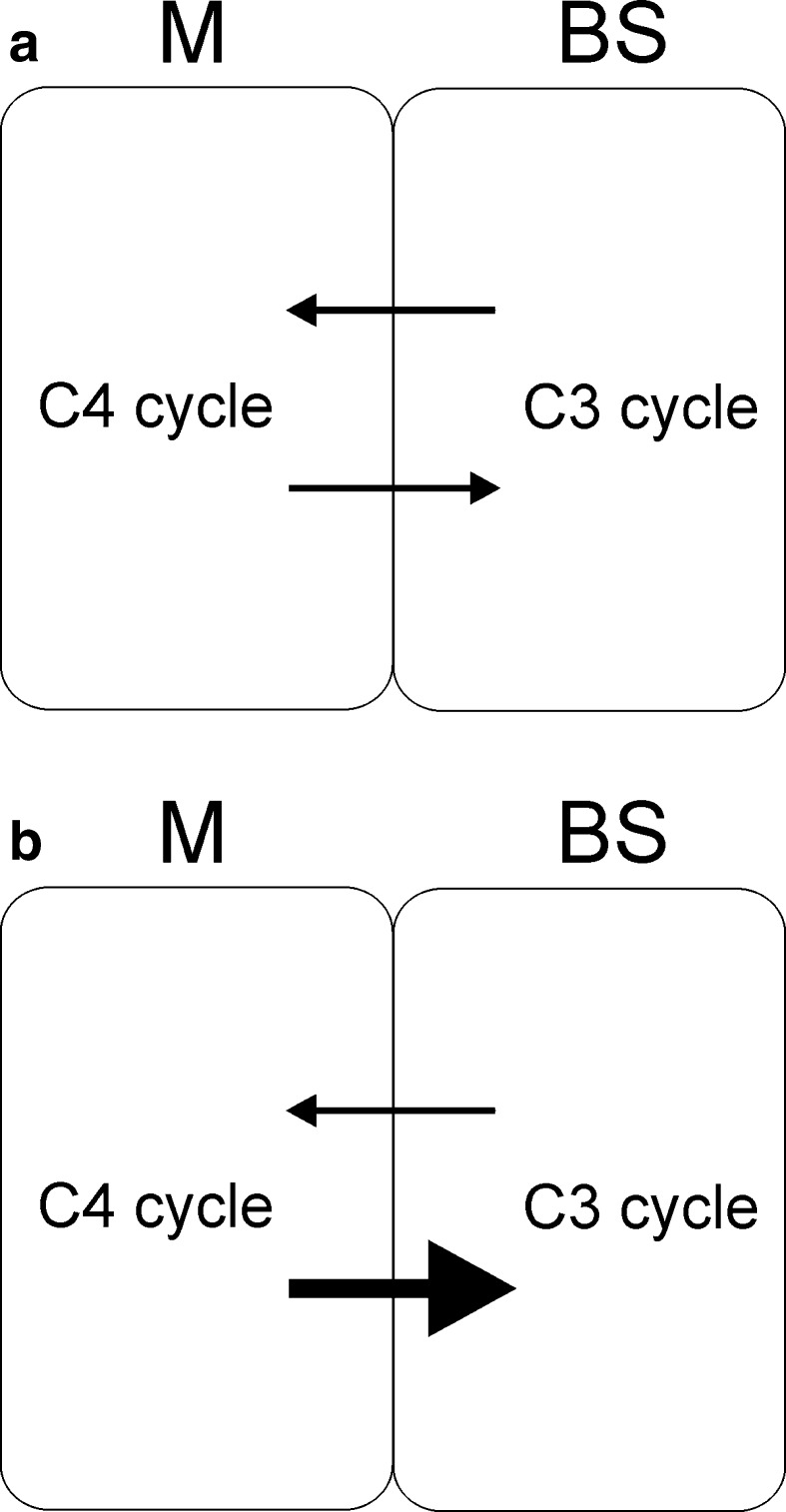
Schematic representation of the metabolite transport between mesophyll (M) and bundle sheath (BS) cells in plants grown under moderate light (**a**) and treated for 1 h with high light (**b**)

Our results showed that the plants grown in identical light conditions responded differently to photoinhibitory light. The most resistant to photoinhibition was the weed plant *E. crus*-*galli* which developed a strategy, allowing to maintain a high photosynthetic activity while protecting the thylakoid proteins from degradation mainly by PSII protein phosphorylation (Fig. 5), high pool of carotenoids (Table 2), and high energization of the leaves (Table 4). The investigated C4 grasses have different granal index of the BS chloroplasts (Ueno et al. 2005). Taking into account that the chloroplast grana are the site where O_2_ is evolved due to PSII activity, the O_2_ and CO_2_ partial pressure values in the BS cells may differ between species. Greatly the reduced grana in BS chloroplasts correlate reasonably well with the differences in PSII activity (Table 3) and simultaneously reduced the BS chloroplasts production of reactive oxygen species (ROS) (Doulis et al. 1997). Also there are differences in the suberization of the BS cell walls. There is considerable heterogeneity in sheath cell development and suberin composition, both within and between grass taxa (Mertz and Brutnell 2014). It probably prevents the leakage of CO_2_ from these cells in a different way. In case of NADP-ME species, the decreased rate of grana formation in BS, and increased malate transport together with decarboxylation, can provide compensatory reducing power (Hatch 1987). Thus, the amount of grana in BS is a specialized feature whose increased demands are satisfied by co-operation between the two cell types and induction of metabolic changes or the changes in the transport of metabolites. The measurements of photosynthesis and respiration rates (Figs. 1, 2) show lower CO_2_ exchange rate in the leaves of *Z. mays* compared to *D. sanguinalis* and *E. crus*-*galli*, which is possibly caused by the limitation of Calvin cycle reactions in maize. In *Z. mays,* the rate of photosynthesis after HL treatment was significantly lower than that of *D. sanguinalis* and *E. crus*-*galli*. We think that, in maize BS cells, a more rapid release of CO_2_ than fixation by the Calvin pathway could result from the limited reducing potential in the BS chloroplasts, which is likely to occur after a period of ML treatment. This may indicate that in BS cells of the investigated plants the assimilatory capacity differs and it is inadequate when the light transition is of short duration. We propose that the rate of C4-cycle reactions, moving metabolites from M to BS cells, is faster during the short-term duration of the HL then in the ML and the Calvin cycle reactions can’t utilize them (Fig. 7). This suggests that according to the capacity of the BS chloroplasts to produce O_2_ and the reducing power, the related differences may occur in the potential of photosynthesis in these grasses.

Previously, it was shown that maize BS cells have a relatively high conductance to CO_2_ which could result in CO_2_ level around Rubisco (Dai et al. 1995). Other C4 species also show differences in conductance to CO_2_ in BS cells (Brown and Byrd 1993). The observed changes might influence the CO_2_ assimilation rate. In *E. crus*-*galli*, the higher rate of photosynthesis and respiration correlated well with higher amount of ATP as shown in Table 4. The CO_2_ assimilation correlated with the electron transport rate (ETR), and the observed differences are found, for the investigated species, in both, the growth conditions (ML) and after treatment of leaves with the high light (ML → HL) (Fig. 4). Substantial differences in the ETR were found between *Z. mays* and *E. crus*-*galli,* indicating a severe limitation in the electron transport in the HL-treated maize leaves. Chlorophyll *a* fluorescence at room temperature is widely used to probe the photosynthetic functions and regulations of plants in vivo (Schreiber 1983). Photochemical quenching (qP) is generally thought to reflect the rate of electron transport and hence photosynthetic flux, whereas non-photochemical quenching (NPQ) reflects heat dissipation of the excitation energy in the antenna system (Baker 2008). In M of C4 plants, where Rubisco is absent, the qP reflects electron transport more accurately than that in C3 chloroplasts. Also, the lower level of PSII abundance in BS chloroplasts means that the whole leaf variable fluorescence emanates almost exclusively from the M thylakoids, which simplifies the interpretation of the signal. The photochemical quenching (qP) (Fig. 3) in the leaves of *E. crus*-*galli* after HL treatment decreased slightly since the photosynthesis rate (Fig. 1) after photoinhibitory light treatment could rapidly reach values similar to the values of the control plants. Recovery of photosynthesis in *E. crus*-*galli* leaves observed after photoinhibition could be related to acclimation that reduces the influence of the excess light in thylakoids without a negative effect on the CO_2_ assimilation rate. The decrease in the qP in *E. crus*-*galli* leaves under HL correlates with the increased dissipation of non-radiative energy (NPQ) and with a higher pool of antheraxanthin and zeaxanthin (A + Z) in the investigated plants. However, the total pool size of carotenoids and the proportion of the photoconvertible violaxanthin are found to be similar in all the investigated species (Table 2). Depending on the available light irradiation, plants have the possibility to adapt to high- or low-light condition. They can switch their photosynthetic apparatus between light harvesting and excess energy dissipation. Many observations indicate that the interactions between chlorophylls and carotenoids seem to be crucial for the excess energy dissipation (Holleboom and Walla 2014). It is known that there are differences in the efficiencies of energy transfer between carotenoids and chlorophylls for different species as well as light-harvesting complexes, but the explanation of this phenomenon is not yet satisfactory (Ritz et al. 2000).

Changes in the NPQ among species are probably attributable to differences in photosynthetic capacity, and the observed lower values of the qP in HL cannot be explained only by increased heat dissipation. The increased NPQ in different investigated species exposed either to ML or to HL may occur due to species-specific response of plants to light and also to other photochemical differences induced by M and BS thylakoid protein composition. Similarly, the amount of Z + A (zeaxanthin + antheraxanthin) in the NADP-ME C4 species can confirm the observation that Z (zeaxanthin) is not a prerequisite for NPQ, because significant levels of NPQ may be induced in the absence of zeaxanthin (Noctor et al. 1991). Our results indicate that possible causal links between zeaxanthin and the NPQ are less obvious in C4 plants than expected. Further studies of this subject are need.

The phosphorylation of LHCII proteins is controlled by the redox state of the plastoquinone pool (Allen 2002). Modulation of light intensity provides a model system for studying the regulation of thylakoid membrane proteins phosphorylation in M and BS chloroplasts and in relation to its structure and function. The changes in the ATP/ADP ratio in leaves also indicate whether any modulation of PSII proteins phosphorylation occurs. For example, a high ATP/ADP ratio in *E. crus*-*galli* leaves in control conditions probably favors protein phosphorylation, in contrast to maize and *D. sanguinalis* (Table 4). However, exposure to HL resulted in a decreased ATP/ADP ratio by about 15–20% in all species, affecting the activity of key C4 enzymes (Nakamoto and Young 1990; Tazoe et al. 2008) and, in effect, the CO_2_ assimilation rates. In case of *E. crus*-*galli* leaves, upon changing photon irradiance from ML to HL, photosynthesis rate decreased and then returned to the control level during 2 h of recovery period (Fig. 1) indicating a high efficiency of the photosynthetic machinery.

It is by now relatively well documented that chloroplasts display a broad range of plasticity regarding the stacked and unstacked thylakoid formation as an adaptation to environmental changes (Maxwell et al. 1999). It was shown previously that there is a different resistance mechanism to various stress factors in M and BS chloroplasts. Particularly, in the M chloroplasts of maize, the photosynthetic output was inhibited as a result of the granal stacking damaged after NaCl treatment, while in the BS chloroplasts, it was increased (Hasan et al. 2006). Results similar to those of maize were obtained by Omoto et al. (2009) for other seven C4 NADP-ME species. The NADP-ME grasses are good model organisms to compare the responses to different stress factors, because of the two types of chloroplasts, mesophyll (rich in PSII) and bundle sheath (rich in PSI), with different redox statuses and ATP contents. Our results confirm that BS chloroplasts lacking grana are deficient in PSII, but they contain an active PSI. The high light stress does not affect the photochemical efficiency of PSII in *E. crus*-*galli* as much as in *Z. mays*, and only a small drop was observed in the measured *F*v/*F*m ratio (maximal efficiency of PSII) (about 10%) (Table 1). These results are well in agreement with the observation showing that the PSII activity (Table 3) was not significantly affected by HL exposure of thylakoids isolated from both the *E. crus*-*galli* and *D. sanguinalis* BS chloroplasts, where the photoreduction of dichlorophenol-indophenol (DCPIP) rate remained unchanged. Contrary to that, HL stress had an inhibitory effect on the electron transport through PSII in BS thylakoids of *Z. mays*. The decreasing activity of PSII was probably related to damages occurring, due to stress, on the donor side of PSII. To investigate the possible effect of HL stress on the organization of the major protein complexes in the thylakoid membrane from M chloroplasts, we employed blue-native (BN) electrophoresis (Fig. 6). In this assay, the control (ML) protein profiles and the HL-treated leaves were clearly similar, which demonstrated that 1 h of HL does not disturb the organization of thylakoid complexes, although it has an inhibitory effect on the photosynthesis rate. This stands in agreement with the fluorescence measurements and PSII activities. The results showed that the short-term effect of HL irradiation causes a decrease in fluorescence, photochemical activity of PSII and PSI, as well as the energetic status of the leaves. However, this two-cell-type system allows to maintain a high photosynthetic activity. Probably, the crucial mechanism, which is involved in the metabolism and regulation/stabilization of protein structure in stress conditions, is a reversible protein phosphorylation. The regulation and physiological significance of phosphorylation/dephosphorylation in both chloroplasts of NADP-ME type C4 species are related to the supramolecular organization of the thylakoid membranes/complexes during photoinhibition.

High light significantly enhanced the phosphorylation of the D1 protein in both types of chloroplasts and induced dephosphorylation of LHCII (Fig. 5). We detected the highest phosphorylation level of the D1 protein in M and BS thylakoids of *E. crus*-*galli,* which suggests a better protection of PSII against photoinhibition compared to *D. sanguinalis* and *Z. mays*. It was found earlier that the phosphorylated form of D1 was resistant to degradation (Rintämaki et al. 1996). Moreover, the HL which induces photoinhibitory effects did not cause any changes in the total amount/content of D1protein (Fig. 5b) in all the examined species. These results correlate well with the photochemical activities. In addition, the different phosphorylation level of PSII proteins (Fig. 5a) and changes in the PSI-LHCII supercomplexes (Fig. 6) indicate different redox states not only in both types of chloroplast but also between investigated plants. Different phosphorylation of LHCII in M and BS chloroplasts and among the C4 species in ML and HL might be the mechanism by which the C4 plants regulate the distribution of excitation energy to both photosystems and maintain the proper function of photosynthetic machinery.

Chloroplasts are plant organelles that are most sensitive to various stress factors because they are the major sources of ROS production in plants cells (Asada 2006). Due to minimal activity of PSII in the BS chloroplasts of NADP-ME species, the ROS production is largely limited and much lower than that in the M chloroplasts. Hence, the M chloroplasts as compared to the BS are more sensitive to oxidative stresses induced by various factors (Foyer et al. 2002; Omoto et al. 2009). The two photosynthetic cell types of the NADP-ME species in accordance with its structure and function help in the efficient photosynthesis even during irradiation stress. To maintain continuously the C4 cycle, energy must be generated photochemically and the coordinated transport of metabolites must take place. Various mechanisms are involved in sustaining the homeostasis in leaves. High light stress has different effects on the rate of photosynthesis and respiration among the selected three species. The antioxidant system in all the species reacted differently after exposure to HL treatment (Tables 5, 6). Activities of superoxide dismutase (SOD) were found to be increased in the leaves of *E. crus*-*galli* and *D. sanguinalis,* whereas in maize they remained unchanged. The activity of ascorbate peroxidase (APX) increased in *D. sanguinalis*, decreased in maize, and remained unchanged in *E. crus*-*galli*. Malondialdehyde (MDA) levels increased in all plants and the H_2_O_2_ amount decreased after HL treatment. *E. crus*-*galli* showed a slight increase in the MDA content (Table 5) compared to *Z. mays*, which indicates that this might be due to the higher content of saturated fatty acids in its lipid membranes. It is, therefore, possible that another adaptation mechanism of the NADP-ME plants to stress conditions may proceed via variations in their fatty acid composition of the lipid membrane. The H_2_O_2_ plays a dual role in plants. At low concentrations, it acts as a secondary signaling molecule involved in the acclimatory signal triggering and raising a tolerance level to various stresses. On the other hand, H_2_O_2_ accumulation gives rise to membrane lipid peroxidation when sufficiently high concentrations are attained (Foyer and Noctor 2005). Our results indicated that the HL treatments did not induce oxidative stress, which was confirmed by the H_2_O_2_ and MDA levels in the leaves of C4 plants, and increased concentrations of MDA did not induce membrane damage. Likely, H_2_O_2_ that was generated was scavenged efficiently enough. The observed decrease in the photosynthesis rate of maize is likely related to changes in M chloroplasts due to differences in light saturation of both types of chloroplasts. The higher light intensity was permeating the M chloroplasts better than the BS chloroplasts, according to the light penetration profile (Zienkiewicz et al. 2015). Therefore, we are inclined to conclude that the differences in the reaction of NADP-ME species to HL treatment are due to the different photon absorption rate by chloroplasts, located in particular type of tissues.

We found that both types of leaves of *E. crus*-*galli* and *D. sanguinalis* grown under ML fixed and released CO_2_ before photosynthesis (*R*_0_) at a similar rate, whereas respiration after Pn (*R*_1_) was different. The leaves of *E. crus*-*galli* showed higher rate of CO_2_ evolution than *D. sanguinalis* (Fig. 2), suggesting that the increased level of CO_2_ evolution might be due to enhanced utilization of malate. Malate can be used, besides its role in photosynthesis, in mitochondrial respiration, providing a likely explanation for the high ATP/ADP ratio observed in *E. crus*-*galli* leaves. We conclude that high efficiency of PSII, higher adenylate pool and PSI and PSII activity after the HL treatment, as well as stronger phosphorylation status of PSII proteins can play a decisive role in granting a better resistance to photoinhibition in *E. crus*-*galli* compared to the other species. In *E. crus*-*galli* leaves, the pool size of the xanthophylls cycle components was slightly higher than for *Z. mays* and *D. sanguinalis*. The percentage of photoconvertible violaxanthin (V) was very similar among all the three species and also between the M and BS thylakoids. Additionally, the zeaxanthin and antheraxanthin (Z + A) content increased after the HL treatment in all the analyzed species. Thus, it seems that a reduced rate of CO_2_ assimilation in HL for maize leaves was likely not related to changes in the content of xanthophyll cycle pigments. Their levels were almost the same as in *D. sanguinalis* where Pn after the HL treatment was higher. The decreased PSII efficiency (*F*v/*F*m ratio) observed for maize leaves in HL could be related to the limitation of carbon supply in BS or qI quenching related to photoinhibition without damaging of D1 protein (Fig. 5b). The drastic decrease in CO_2_ uptake in maize leaves after exposure to HL treatment could be a simple effect of a high decrease in photosystems activity caused by photoinhibitory light. We also suggest that the adenylate pool found similar in all light conditions in maize and *D. sanguinalis* was not the major limiting factor for CO_2_ assimilation. It seems that in maize the CO_2_ uptake controls the PSII efficiency. The light intensity which penetrates into the leaves may control the level of phosphorylation of PSII proteins and thus photoinhibition. The differences in the PSI and PSII activity in chloroplasts of both cell types of C4 species indicate that these plants have different ratios of energy production in cyclic and noncyclic electron flow, stemming from different capacity to produce sufficient amounts of NADPH and ATP needed for fixing CO_2_.

### **Author contribution statement**

ER conceived this study. ER and MZ designed and evaluated the experiments. AB, AD, TK, EP, PR, WW, and MZ performed the experiments. ER wrote the manuscript.

## Abbreviations

APX: Ascorbate peroxidase
BS: Bundle sheath
ETR: Relative electron transport rate
*F*v/*F*m: Ratio of variable to maximum chlorophyll fluorescence
HL: High light
LHCII: Chlorophyll *a*-/*b*-binding protein of photosystem II
M: Mesophyll
MDA: Malondialdehyde
ML: Moderate light
NADP-ME: NADP-depended malic enzyme
NPQ: Non-photochemical quenching
SOD: Superoxide dismutase
